# From past to present: tracing the trends of diabetes drug trials in mainland China

**DOI:** 10.3389/fendo.2024.1427148

**Published:** 2025-01-10

**Authors:** Zengqing Ma, Xin Zhao, Yu Lin, Hong Zhang, Lianping Wu, Yang Tao, Hongjun Shi, Susu Li

**Affiliations:** ^1^ Department of Pharmacy, Nanjing Gaochun People’s Hospital, Nanjing, Jiangsu, China; ^2^ Clinical Trial Center, Nanjing Gaochun People’s Hospital, Nanjing, Jiangsu, China; ^3^ Department of Respiratory Medicine, Nanjing Gaochun People’s Hospital, Nanjing, Jiangsu, China

**Keywords:** diabetes, drug clinical trials, research and development, trends, mainland China

## Abstract

**Background:**

This study aimed to analyze the changing trend of diabetes drugs clinical trials in China during 2013-2023, and provided a reference for the research and development of diabetes drugs.

**Methods:**

Diabetes drug clinical trial data were obtained from the registration and information disclosure platform of the National Medical Products Administration (NMPA) between January 1, 2013, and December 31, 2023. Trends of clinical trials on diabetes drugs were systematically analyzed in terms of characteristics, trial design, time trends, drug type, and indications.

**Results:**

From 2013 to 2023, a total of 1,256 diabetes drugs clinical trials have been registered on the NMPA platform, of which 1056 were chemical drugs and 184 were biological products. The indications are mainly type 2 diabetes mellitus (n=1237, 98.49%). Among them, 838 clinical trials have been completed, 379 were proceeding, and 39 have been terminated or suspended. There were 42 international multi-center clinical trials, and the remaining 1034 clinical trials were domestic. Bioequivalence trials were 691, accounting for 55.02%, followed by 340 phase I clinical trials and 169 phase III clinical trials. The leading units were mostly distributed in eastern China. The proportion of clinical trial sponsorship from domestic pharmaceutical companies is higher than that from overseas companies.

**Conclusions:**

China has made significant advancements in diabetes drug research and development over the past decade. However, problems such as serious drug homogeneity, and insufficient innovation have become increasingly prominent. The government, clinical trial institutions, and pharmaceutical companies must collaborate to promote the high-quality development of drug clinical trials.

## 1 Introduction

Presently, diabetes ranks among the most common chronic metabolic diseases in the world. Its symptoms are reflected in the increase of blood sugar caused by a variety of causes, with an estimated impact on 693 million adults by 2045 (1, 2). In China, more than 110 million people suffer from diabetes (3), with a high incidence rate of 12.8% (4). The prevention and treatment of diabetes remains a long and arduous task. Microvascular complications were the main causes of morbidity in diabetes patients, which greatly affected their quality of life (5, 6). What’s more, diabetes costs $966 billion a year in health expenditures worldwide and causes more than 6.7 million deaths each year, placing a huge burden on public health (7). Given the dangers of diabetes and its complications, the priority of research and development (R&D) of related therapeutic drugs has gradually increased.

China is the largest clinical trial market in Asia, followed by India and Southeast Asia (8). Global pharmaceutical companies are particularly interested in the Asian market due to the large patient population, fewer competing trials, and rapid patient recruitment. Additionally, the cost of managing clinical trials is relatively low. Since the 1990s, with the robust development of the Chinese economy, clinical research facilities have been continuously improving (9, 10). Drug approval processes in China were plagued with backlogs and delays before 2013, commonly called "drug lag" (11, 12). Against this backdrop, the NMPA established the Registration and Information Disclosure Platform for Drug Clinical Trials in 2013 and issued “Opinions on Continuing the Reforms in Drug Review and Approval and Further Encouraging Pharmaceutical Innovation” to accelerate the approval process and encourage drug innovation (13, 14). In July 2015, the NMPA issued an “Announcement on Carrying out Self-inspection and Verification of Drug Clinical Trial Data” (15, 16). Based on the principles of “the most rigorous standards, the strictest supervision, the most severe penalties, and the most serious accountability”, the sponsors were required to conduct self-inspection of trial data. For drug clinical trials with inauthentic and incomplete data, the registration applications need to be withdrawn by their sponsors. By rectifying the trend of falsification, this announcement ensured the authenticity of clinical trial data and improved the transparency of the approval process.

In 2017, the NMPA relaxed restrictions on the approval of imported drugs, allowing clinical trial data acquired abroad could be accepted (17, 18). This significant countermeasure issued by NMPA encouraged foreign pharmaceutical companies to carry out clinical trials simultaneously, making it more feasible for China’s participation in international multi-center diabetes drug clinical trials. Following its recruitment into the International Council for Harmonization of Technical Requirements for Pharmaceuticals for Human Use (ICH) as a regulatory member, the NMPA established a working office within a month (19). And it was elected as a member of the ICH Management Committee one year later. By acquiring knowledge of the latest international regulatory concepts and advancements, China’s regulatory capacity and level of drug clinical trials were further improved. These efforts gradually brought Chinese drug clinical trials following international standards. Currently, China is accelerating the approval of innovative therapies and devices, and non-Chinese biopharmaceutical companies are benefiting from new regulations. As a result, international collaborative clinical trials of diabetes drugs are common in mainland China nowadays.

The R&D and production of therapeutic drugs were important indicators to measure a country's economic and scientific capacity (20). The Chinese government has shown increasing interest in investing in the clinical trial industry to narrow the gap with developed countries (21–23). Through successive regulatory policies introduced since 2015, the Chinese government has given clinical trials a broader space and platform and guaranteed diabetes drug R&D (24–26). Since then, the registration of clinical trials of various drugs has been increasing every year, and the number of innovative drugs related to diabetes has also been added (27). With the arrival of the era of globalization, drug development, and registration have entered a new chapter, and methods for analyzing drug clinical trials have certain guiding significance in analyzing diabetes drug R&D in China in the present day, amount and distribution of clinical trials in medical institutions, selection of research centers, and patient enrollment. This survey might be useful for sponsors and stakeholders to determine development strategies.

As a result, drug clinical trials are receiving growing attention from both the Chinese government and the pharmaceutical industry. In this study, we systematically reviewed the landscape of diabetes drug clinical trials registered on the National Medical Products Administration (NMPA) platform from 2013 to 2023. It is hoped that the analysis and prospect of this study on the current situation of diabetes drug R&D can provide a theoretical basis and data support for the research and application of new drugs.

## 2 Methods

### 2.1 Eligibility criteria

To ensure the accuracy and reliability of the results of a clinical trial, the following criteria must be met (1): The indications were diabetes mellitus. (2) The year of registration was 2013-2023. Exclusion criteria were as follows: (1) Clinical trials that were registered repeatedly. (2) The sites for the study weren’t located in mainland China. (3) Unrelated drug indications. (4) Missing important information, such as the name of the trial drug, the type of drug, and the name of the principal investigator (PI).

### 2.2 Information sources

Clinical trial data of diabetes drugs passed the National Medical Products Administration (NMPA) Drug Clinical Trial Registration and Information Disclosure Platform (http://www.chinadrugtrials.org.cn). The National Food and Drug Administration established the platform in 2013 to strengthen clinical trial supervision. All registered drug clinical trials must register on the platform, and trials initiated before 2013 but not completed must be registered retrospectively. This makes the NMPA registration platform representative and authoritative in the Chinese clinical trials market. With this in mind, 2013 was determined as the start year of the study.

### 2.3 Search strategy

From January 1, 2013, to December 31, 2023, a total of 23040 drug clinical trials had been registered on the NMPA platform. To comprehensively cover drug trials in the category of diabetes, we conducted an independent search for “Type 1 Diabetes Mellitus” and “Type 2 Diabetes Mellitus” as keywords.

### 2.4 Selection process

**Figure 1:**
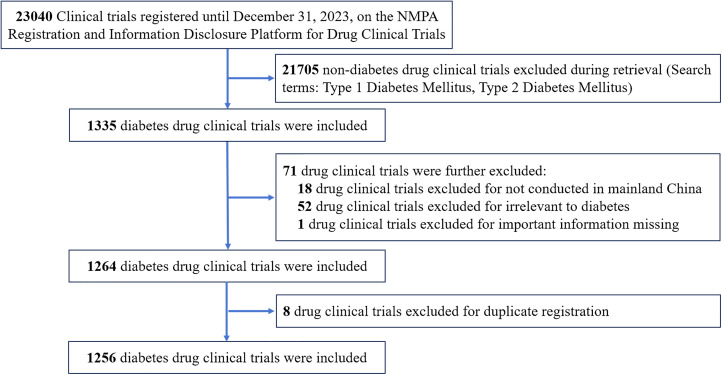
Flow diagram of study retrieval and screening.

For inclusion of eligible clinical trials, two authors (Z Ma and X Zhao) searched the NMPA platform through the two keywords above, and 1335 drug clinical trials were identified. To determine the final analytical data set, three authors (Y Lin, H Zhang, and L Wu) independently reviewed the registration information for the title, indication, registration ID, study design, and study location of the 1335 drug clinical trials, and excluded trials that did not meet the above criteria. Then, another three authors (Y Tao, H Shi, and S Li) identified duplicated trials. Finally, 79 clinical trials were excluded and 1256 clinical trials were included. The data retrieval and filtering process is shown in **Figure 1**.

### 2.5 Data item and extraction

The data, including time, location, dosage forms, indications, trial stages, sponsors, and institutions, were extracted from the included drug clinical trials. Two authors (Z Ma and S Li) collaborated to compile all available information in a Microsoft Excel spreadsheet. Quantitative variables were represented by numbers and percentages for descriptive analysis. Data statistics and analysis were performed using Microsoft Excel 2016 and GraphPad Prism 9.0.

## 3 Results

### 3.1 Characteristics of Drug Clinical Trials

From 2013 to 2023, a total of 1335 clinical trials on diabetes drugs were identified in the NMPA platform conducted in China, and ultimately, 1256 clinical trials were included in the study. The completion status of these clinical trials was considered. As shown in **Table 1**, 838 (66.72%) clinical trials had been completed and 379 (30.18%) clinical trials were in progress. In addition, 22 (1.75%) clinical trials were suspended and 17 (1.35%) clinical trials were in the status of termination. In terms of the indications, type 2 diabetes mellitus (T2DM) was the most common, accounting for 1237 trials (98.49%), while type 1 diabetes mellitus (T1DM) accounted for only 7 trials (0.56%). Additionally, a total of 12 trials (0.96%) were indicated for both T1DM and T2DM. Crossover studies accounted for the largest proportion (780, 62.10%), followed by parallel group studies (414, 32.96%) single-arm studies (61, 4.86%), and factorial design (1, 0.08%). In terms of randomization, there were 1171 randomized trials (93.23%) and 85 non-randomized trials (6.77%). In terms of blind design, there were 970 open-label trials (77.23%), 277 double-blind trials (22.05%), and 9 single-blind trials (0.72%). What’s more, domestic trials were still the mainstream in the field of diabetes across mainland China, with a total of 1214, accounting for 96.66%, while there were 42 international multi-center clinical trials (3.34%) in the past decade.

**Table 1:** Characteristics of cardiovascular drug clinical trials in China.

Characteristics	Number of clinical trials (%)
Status	
Completed	838 (66.72%)
Proceeding	379 (30.18%)
Suspended	22 (1.75%)
Terminated	17 (1.35%)
Indication	
T1DM	7 (0.56%)
T2DM	1237 (98.49%)
T1TD & T2DM	12 (0.95%)
Design	
Crossover	780 (62.10%)
Parallel-group	414 (32.96%)
Single-arm	61 (4.86%)
Factorial design	1 (0.08%)
Randomization	
Randomization	1171 (93.23%)
Non-randomization	85 (6.77%)
Blinding	
Open-label	970 (77.23%)
Double-blind	277 (22.05%)
Single-blind	9 (0.72%)
Coverage	
Domestic trial	1214 (96.66%)
IMCT	42 (3.34%)

T1DM, type 1 diabetes mellitus; T2DM, type 2 diabetes mellitus; IMCT, international multi-center clinical trials.

### 3.2 Geographical distribution of drug clinical trials

**Figure 2:**
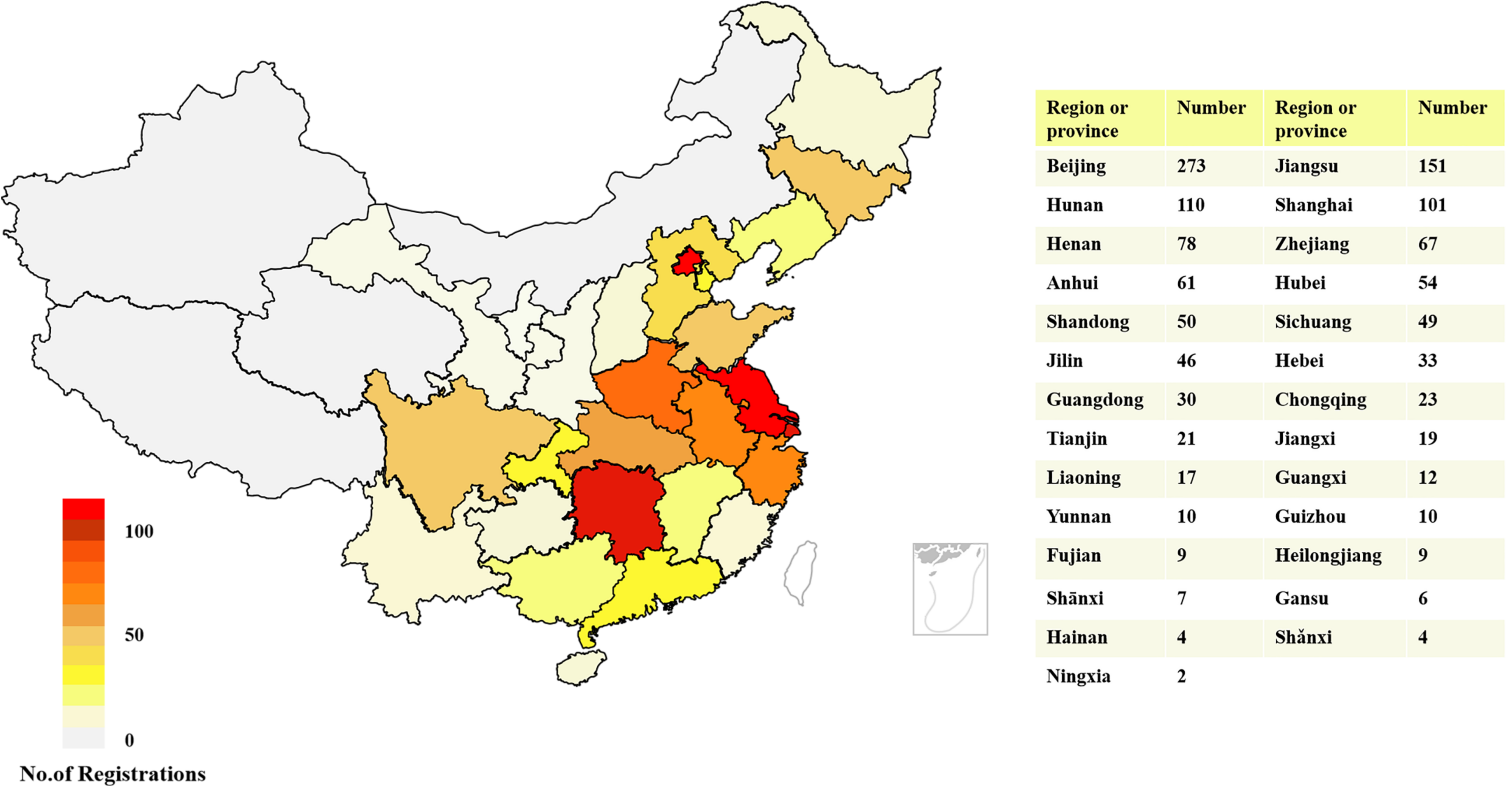
Geographical distribution of diabetes drug clinical trials according to leading units in China, 2013–2023.

In general, the leading units of these 1256 diabetes drug clinical trials were distributed in 27 provinces across China. A total of 273 leading units of clinical trials for diabetes drugs were initiated in Beijing, accounting for the highest proportion (21.74%), followed by Jiangsu (n = 151) and Hunan (n = 110), with proportions of 12.02% and 8.76%, respectively. In contrast, the number of clinical trials whose leading units from Hainan (n = 4), Shănxi (n = 4), and Ningxia (n = 2) was less than five. In addition, there are 4 provinces and regions without leading units, reflecting a severely uneven geographical distribution of diabetes drug clinical trials across mainland China. The detailed geographic distribution of clinical trials is illustrated in **Figure 2**.

### 3.3 Distribution of drug types

**Figure 3:**
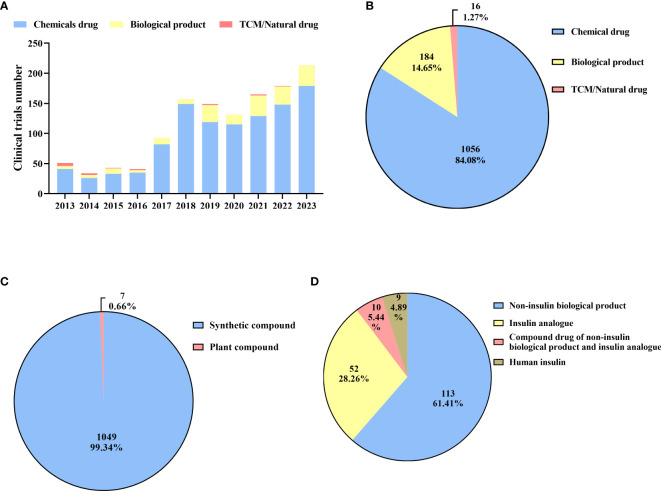
Distribution of drug types **(A)**: Annual numbers of initiated diabetes drug clinical trials by drug types in China, 2013–2023; **(B)**: Drug types of diabetes drug clinical trials; **(C)**: Types of chemical drugs; **(D)**: Types of biological products.

From 2013 to 2016, the number of clinical trials was relatively low, with fewer than 60 clinical trials per year (**Figure 3A**). The number of clinical trials witnessed a substantial increase in 2017. Since then, the annual number has exceeded 90. It was not until 2023 that the number reached its peak, with 213 clinical trials conducted throughout the whole year. The number of clinical trials witnessed a significant surge from 51 in 2013 to 213 in 2023, representing a remarkable increase of 317.65%. In total, there were 1056 chemical drugs, accounting for the major proportion (84.08%) of diabetes drug clinical trials conducted in mainland China (**Figure 3B**). This included 1049 synthetic compounds and 7 bioactive compounds derived from plants, accounting for 99.34% and 0.66%, respectively (**Figure 3C**). This was followed by biological products, with a total of 184 drug clinical trials conducted, including 113 non-insulin biological products (61.41%), 52 insulin analogs (28.26%), 9 human insulin (4.89%), and 10 compound drugs (5.44%). These compound drugs consist of non-insulin biological products and insulin analogs (**Figure 3D**). Recently, there has been explosive growth in the number of biological product clinical trials, with 142 carried out in the past five years, accounting for 77.17% of all biological product clinical trials. However, the number of clinical trials on traditional Chinese medicine (TCM)/natural drugs has been limited over the past decade, with only 16 conducted, all of which were herbal drugs.

### 3.4 Distribution of phases

**Figure 4:**
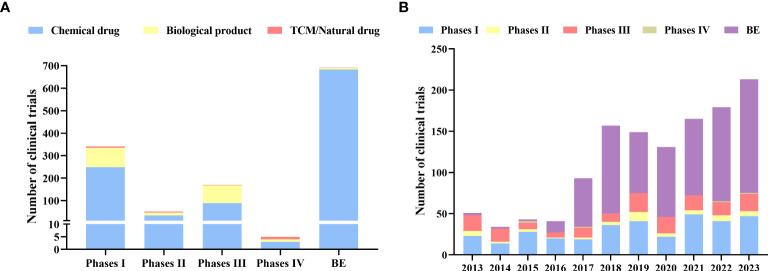
Distribution of phases **(A)**: Distribution of clinical trials by phases; **(B)**: Annual numbers of initiated diabetes drug clinical trials by study phase in China, 2013–2023.

Bioequivalence (BE) studies accounted for more than half (n=691, 55.02%), followed by phase I (n=340, 27.07%) and phase III (n=169, 13.46%). Phase II and phase IV only account for 4.06% and 0.40% respectively (**Figure 4A**).

BE trials have outpaced phase I since 2015 and have shown a dramatic increase between 2015 and 2023 (**Figure 4B**). In particular, there was a substantial 321% increase in the number of BE studies conducted in 2017 compared to the previous year, with a total of 59 clinical trials conducted. There have been more BE studies than phase I studies since 2017. More than 64.67% and 23.48 % of the trials on chemical drugs were conducted at Phase I or BE stages, respectively, while 89.67% of the biological products are undergoing tests at Phase I and III (**Figure 4A**). In contrast, 68.75% of clinical trials for TCM/natural drugs are in phases I and II.

### 3.5 Distribution of dosage forms

**Figure 5:**
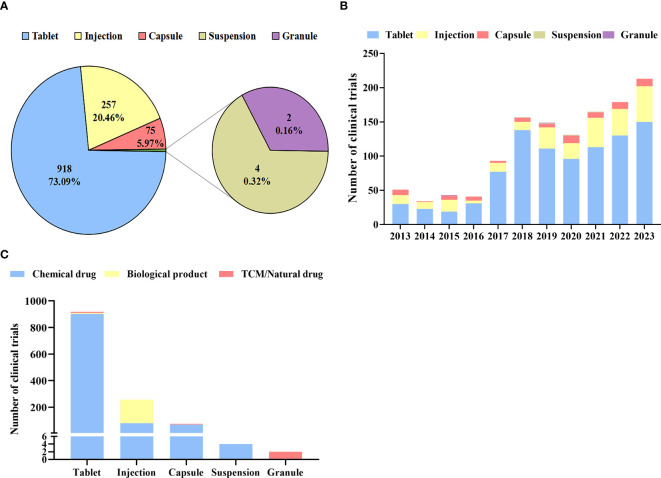
Distribution of dosage form **(A)**: Dosage forms of diabetes drug clinical trials; **(B)**: Annual numbers of initiated diabetes drug clinical trials by dosage forms in China, 2013–2023; **(C)**: Distribution of clinical trials by Dosage forms.

There were a total of 5 dosage forms examined in all clinical trials (**Figure 5A**). tablets were the main dosage form (n=918) in diabetes drug clinical trials in China, accounting for the highest proportion (73.09%), followed by injection (n=257) and capsule (n=75), accounting for 20.46% and 5.97% respectively. In contrast, the number of granules and Suspensions was fewer than five. While other dosage forms increased slowly, the number of tablets grew rapidly from 2013 to 2023, showing a 2.48-fold change in 2017, and reaching a peak of 138 in 2018 (**Figure 5B**).

As shown in **Figure 5C**, chemical drugs, biological products, and TCM/natural drugs have different dosage forms. A majority of chemical drugs were administered as injections, tablets, and capsules (99.62%), with tablets accounting for the majority (85.51%). However, 96.20% of biological products were given as injections. The dosage forms of TCM/natural drugs are diverse, including tablets, capsules, and granules.

### 3.6 Distribution of single agents and compound drugs

**Figure 6:**
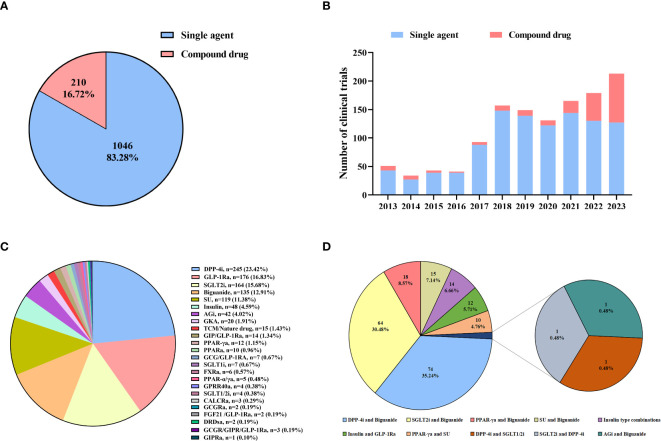
Distribution of single agents and compound drugs **(A)**: Drug ingredients of diabetes drug clinical trials; **(B)**: Annual numbers of clinical trials by drug ingredients; **(C)**: Pharmacological mechanism of single agents; **(D)**: Combinations of compound drugs. DPP-4i, dipeptidyl peptidase-4 inhibitors; GLP-1Ra, glucagon-like peptide-1 receptor agonists; SGLT2, sodium-dependent glucose transporter 2 inhibitors; SU, sulfonylureas; Agi, α-glucosidase inhibitors; GKA, glucokinase activator; TCM, traditional Chinese medicine; PPAR-γa, peroxisome proliferator-activated receptor-γ agonists; PPARa, peroxisome proliferator-activated receptor agonists, SGLT1i, sodium-dependent glucose transporter 1 inhibitors; FXRa, farnesoid X receptor agonists; PPAR-α/γa, peroxisome proliferator-activated receptor-α/γ agonists; GPR40a, G protein-coupled receptor 40 agonists, SGLT1/2i, sodium-dependent glucose transporter 1/2 inhibitors, CALCRa, calcitonin receptor agonists; DRDsa, dopamine receptor genes agonists; GCGRa, glucagon receptor antagonists; GIPRa, Glucose-dependent insulinotropic polypeptide receptor agonists; FGF21, fibroblast growth factor 21.

Based on the drug component, the trial drugs were divided into two classes: single agents and compound drugs. Among all the clinical trials, there were 1046 single agents and 210 compound drugs, accounting for 83.28% and 16.72% respectively (**Figure 6A**). From 2013 to 2020, less than 10 clinical trials of compound drugs were conducted each year (**Figure 6B**). It showed that monotherapy was the mainstream of diabetes drug clinical trials in this period. The situation, however, changed in 2021. The number of clinical trials in 2021 was 21, representing a twofold increase compared to the previous year. Since 2021, number of clinical trials for compound drugs has been increasing year by year, reaching a peak in 2023 with a total of 86 trials conducted throughout the year.

The clinical trials of single agents and compound drugs were further analyzed according to the pharmacological mechanism and more details are shown in **Supplementary Table 1**. In 1246 single-agent clinical trials, 24 hypoglycemic mechanisms detailly were identified (**Figure 6C**). The number of trials in dipeptidyl peptidase-4 inhibitors (DPP-4i) ranked first (n=245, 23.42%), while glucagon-like peptide-1 receptor agonists (GLP-1RA, n=176, 16.83%) and sodium-dependent glucose transporter 2 inhibitors (SGLT2i, n=164, 15.68%) took the second and third place. In terms of compound drugs, there are 10 combinations (**Figure 6D**). The combination of DPP-4i and biguanides was the most common (n=74, 35.24%), followed by the combination of SGLT2i and biguanides (n=64, 30.48%) and the combination of peroxisome proliferator-activated receptor-γ agonists (PPAR-γa) and biguanides (n=18, 8.57%).

### 3.7 Distribution of leading units and sponsors

From 2013 to 2023, a total of 239 hospitals conducted clinical trials on diabetes as leading units. The top 3 leading units by the number of registered clinical trials were Peking University People’s Hospital (n=62, 4.93%), The First Hospital of Jilin University (n=33, 2.63%), and Nanjing Drum Tower Hospital (n=31, 2.47%, **Table 2**). The departments of endocrinology of these hospitals were at the leading level in mainland China.

**Table 2:** Top 10 leading units by the number of registered diabetes drug clinical trials.

Ranking	Leading unit	Number (Percentage)
1	Peking University People's Hospital	62 (4.93%)
2	The First Hospital of Jilin University	33 (2.63%)
3	Nanjing Drum Tower Hospital	31 (2.47%)
4	Shanghai Public Health Clinical Center	30 (2.39%)
5	Xiangya Boai Rehabilitation Hospital	28 (2.23%)
6	China-Japan Friendship Hospital	27 (2.15%)
7	Shanghai Xuhui District Central Hospital	25 (1.99%)
8	Peking University First Hospital	24 (1.91%)
8	Beijing Hospital	24 (1.91%)
8	Henan Zhonghui Hospital	24 (1.91%)

From 2013 to 2023, a total of 479 companies and research institutions applied as sponsors to perform clinical trials on diabetes. Among the 1256 clinical trials, 1160 clinical trials (92.36%) were sponsored by domestic pharmaceutical companies, and 88 clinical trials (7.01%) were sponsored by foreign pharmaceutical companies (**Table 3**). Only 8 clinical trials (0.64%) were sponsored by domestic research institutions.

**Table 3:** Phases of clinical trials by different sponsors.

Sponsors	I	II	III	IV	BE	Total
Domestic pharmaceutical companies	314 (25.00%)	50 (3.98%)	103 (8.20%)	2 (0.16%)	691 (55.01%)	1160 (92.35%)
Foreign pharmaceutical companies	22 (1.75%)	0 (0.00%)	64 (5.10%)	2(0.16%)	0 (0.00%)	88 (7.01%)
Domestic research institutions	4 (0.32%)	1 (0.08%)	2 (0.16%)	1(0.08%)	0 (0.00%)	8 (0.64%)

BE studies were the most common clinical trials conducted by domestic pharmaceutical companies (n=691, 59.57%), followed by Phase I trials, with 314 clinical trials (27.07%). The foreign pharmaceutical companies most frequently conducted Phase III trials (n=64, 72.73%), which were followed by Phase I trials (n=22, 25.00%). Jiangsu Hengrui Pharmaceutical Co., Ltd., had the highest number of registered diabetes drug clinical trials (n=66, 5.25%), followed by China Shijiazhuang Pharmaceutical Group Co., Ltd., (n=32, 2.55%) and Jiangsu Deyuan Pharmaceutical Co., Ltd., (n=23, 1.83%). Among the top 10 sponsors in the number of registered diabetes drug clinical trials, there were 1 foreign and 9 domestic pharmaceutical companies (**Table 4**).

**Table 4:** Top 10 sponsors by the number of registered diabetes drug clinical trials.

Ranking	Sponsors	Number (Percentage)
1	Jiangsu Hengrui Pharmaceutical Co., Ltd.	66 (5.25%)
2	China Shijiazhuang Pharmaceutical Group Co., Ltd.	32 (2.55%)
3	Jiangsu Deyuan Pharmaceutical Co., Ltd.	23 (1.83%)
4	Hangzhou Zhongmei Huadong Pharmaceutical Co. Ltd	21 (1.67%)
5	Eli Lilly Asia representative office in Shanghai	20 (1.59%)
6	Jiangsu Haosen Pharmaceutical Group Co., Ltd.	19 (1.51%)
6	Zhengda Tianqing Pharmaceutical Group Co., Ltd.	19 (1.51%)
8	Jilin Tonghuadongbao Pharmaceutical Co., Ltd.	17 (1.35%)
8	Qilu Pharmaceutical Co., Ltd	17 (1.35%)
8	Guangdong Dongyangguang Pharmaceutical Co. Ltd	17 (1.35%)
8	Zhejiang Huahai Pharmaceutical Group Co., Ltd.	17 (1.35%)

## 4 Discussion

This study reports for the first time on the current status of clinical trials for diabetes medications in China. We summarize relevant information from the past decade regarding clinical trials of diabetes drugs in our country, reflecting the trends and challenges faced in this field. This data can provide pharmaceutical companies with references for making new drug development decisions targeted at the Chinese population. The strength of this study lies in its inclusion of clinical trial information for all diabetes medications in China. It is expected to promote the development of diabetes drugs in our country and provide insights into the latest research trends. This study conducted a systematic review of the diabetes drug clinical trials from 2013 to 2023 in mainland China. During the past decade, the number of diabetes drug clinical trials has increased rapidly, as well as the number of leading units, participating units, and drug types, indicating that China has experienced significant development momentum in diabetes drug clinical trials, with an accelerating speed of drug R&D. It has become increasingly important to address problems such as an unequal geographical distribution of leading units and the homogenization of drugs.

Over the past decade, clinical trials for diabetes drugs have developed rapidly due to Chinese government policies aimed at promoting drug development and innovation (28, 29). The NMPA needed to register and publish information about all approved drug trials. In addition, it required additional registration for previously approved drug clinical trials. Because of this, the number of diabetes drug clinical trials registered in 2013 was higher than in 2014 (**Figure 3A**). Subsequently, in August 2015, the State Council issued “Opinions on Reforming the Examination and Approval System for Drugs and Medical Devices”, further strengthening the government's supervision of drug clinical trials (30). These policies had a significant impact on the reform and upgrading of the quality management systems of pharmaceutical companies and clinical trial institutions. The main objectives of the reform were to improve the efficiency of review and approval to eliminate backlogs. Also, BE studies are required for generic drug approval of drugs and priority review process is granted to medicines with urgent unmet medical needs. Therefore, we ascribed the observed decline in the number of diabetes drug clinical trials in 2016 to this particular situation (**Figure 3A**).

As part of the initiative to encourage pharmaceutical companies to carry out BE studies, the State Council issued "Opinions on the Evaluation of Quality and Efficiency of Generic Drugs" in 2016 (31). Before a generic drug could be approved for marketing, it had to be evaluated for consistency with the original drug through BE studies. Assuring drug quality and encouraging pharmaceutical industry upgrades and adjustments were paramount. As a result, 2016 saw an explosive increase in the number of BE studies. Since then, the mainstream status of BE trials on diabetes drugs has been established (**Figure 4**). Furthermore, a series of issued policies made the approval process for drug clinical trials more standardized and rapid, such as “Opinions on Deepening the Reform of the Evaluation and Approval System and Encouraging the Innovation of Pharmaceutical and Medical Devices” and “Good Clinical Practice (GCP)” (32, 33). Therefore, there has been a golden period for diabetes drug clinical trials since 2017, with hundreds of trials being conducted each year (**Figure 3A**).

A greater number of clinical trials were conducted on diabetes drugs that are new to this field. Inhibitors of the enzyme DPP-4, such as sitagliptin, lower blood sugar while being safe and tolerable. Vildagliptin and alogliptin were well tolerated and showed excellent safety profiles. The drugs are a promising first-line treatment option for patients in Asia with T2DM. Liraglutide is a well-established hypoglycemic agent with a good safety profile (34, 35). Research into current hypoglycemic drugs, which have the potential to become the first-line drug for diabetes in the future, is receiving increased attention.

Drug combination therapy has distinct advantages over monotherapy in the treatment of diabetes, including reducing treatment burden, improving efficacy, increasing patient satisfaction, reducing treatment burden, and enhancing treatment adherence (36, 37). Therefore, compound drugs are becoming increasingly popular worldwide. In our study, the results showed that 17.72% of trial drugs for diabetes in mainland China were compound drugs, with an increasing number annually (**Figure 6B**). According to the administration route, we divided the compound drugs into two types. The first type is compound injections, which are usually mixtures of different types of insulin or insulin analogs in specific proportions. For example, an insulin as part 30 injection is a combination of 30% insulin aspartate and 70% insulin protamine aspartate. The second type is compound oral drugs, which are combinations of hypoglycemic agents with different pharmacological mechanisms. Until 2023, a total of 184 clinical trials on compound oral drugs were carried out, accounting for 14.65% of the whole diabetes drug clinical trials. Metformin is the most common ingredient in compound oral drugs. Currently, metformin is the first-line treatment for diabetes and the most commonly prescribed drug for diabetes in the world (38). It can improve control of blood glucose levels without causing weight gain or hypoglycemia. However, all compound oral drugs for clinical trials in mainland China were exclusively dual therapies, with no clinical trials conducted on triple-drug combinations.

There is no doubt that China has not reached the international leading level in the field of compound diabetes drug R&D. In 2019, the first triple combination regimen was approved by the US FDA, consisting of Dapagliflozin, Saxagliptin, and Metformin. The results of the Phase III clinical trial showed that triple therapy improved blood glucose control was well tolerated, and had a lower incidence of hypoglycemia. The results of the Phase III clinical trial showed that the triple combination regimen performed well in glycemic control and tolerance, and had a lower incidence of hypoglycemia (39). What’s more, another triple combination of linagliptin, metformin, and empagliflozin was approved by the US FDA in 2020. Given the lack of triple combination hypoglycemic drugs in mainland China, relevant investment and R&D need to be further strengthened.

The regional distribution of clinical trials of diabetes drugs in the Chinese Mainland is obviously unbalanced, and the number of leading units in the eastern region is significantly more than that in the western region (**Figure 2**). This is consistent with the findings of Su X et al. regarding trends in innovative drug development in China (40). 239 leading units are all experienced hospitals. Therefore, in our view, it was the uneven distribution of superior medical resources for clinical research rather than the uneven distribution of population or patients that contributed to the geographical disparity. This might be attributed to the Chinese government's prioritized resource allocation to major hospitals, which were required to play a leading role. To allocate medical resources more rationally, China implemented several healthcare reforms, including the hierarchical diagnosis and treatment system, the medical community, and telemedicine. Over time, we believed that these reform measures would have a satisfactory effect.

Our study found that 44.77% of leading units conducted only BE studies. This result indicated that some clinical trial institutions lacked the experience to participate in multi-center clinical trials or the registration of new drugs, suggesting that there was a lack of high-level research capabilities in these clinical trial institutions. In addition, there were more than 300 hospitals registered as Phase I clinical trial specialties in mainland China, yet only 110 institutions conducted Phase I diabetes drug clinical trials. This indicated that qualified PI were not highly motivated to carry out Phase I clinical trials. These phenomena suggested that drug clinical trial institutions did not make full use of superior medical resources in mainland China, consistent with a previous report (41, 42). To increase the enthusiasm of physicians to participate in clinical trials and make full use of the medical resources of clinical trial platforms, China issued the “Work Requirements for the Construction Project of Clinical Evaluation Technology Platforms for New Drugs” in 2019, which further stipulated the organizational structure, staffing, process management, and hardware and software facilities of drug clinical trial institutions (43). To encourage PIs to participate in clinical trials, clinical trial projects were included in the evaluation of research performance according to the policy, which were regarded as financial research projects. Through these measures, the utilization degree and utilization efficiency of superior medical resources might be improved in the near future.

Nowadays, there remains a gap between domestic and foreign countries in the R&D of diabetes drugs (44, 45). The majority of clinical trials for diabetes drugs were occupied by BE trials, and there was a serious duplication of research, resulting in limited diversity in innovative drug development. In the study, we found that there were only 168 different drugs involved in the 1256 diabetes drug clinical trials, with an average of 7.48 clinical trials per drug (**Supplementary Table 1**). A total of 135 drug clinical trials were registered on different dosage forms of metformin, followed by empagliflozin and sitagliptin with 54 and 46, respectively. In addition, further statistics show that there are as many as 331 clinical trials involving metformin-related single or compound agents. A large number of meaningless and repetitive clinical trials in mainland China have adversely affected diabetes drug innovation, eroding the R&D environment. This might be due to the large investment, long return cycle, and high risk of innovative drug clinical trials, which have caused many Chinese pharmaceutical companies to focus on mature diabetes generic drugs.

By analyzing data from mainland China's only mandatory clinical trial registration platform, we conducted a systematic analysis of the landscape of diabetes drug clinical trials in mainland China from 2013 to 2023. However, there were some limitations in this study. First, the manual retrieval and screening of diabetes drug clinical trials might introduce potential selection bias. This bias could arise from various factors such as the expertise and experience of the individuals involved in the retrieval process, and any preconceived notions they might have about certain drugs. Secondly, although multiple hospitals were listed as co-leading units in some drug clinical trials, we only take the unit that ranks first as the leading unit for each trial. Thirdly, the pharmacological mechanisms of TCM/Natural drugs were completely different from those of chemical drugs. Therefore, when we statistical the pharmacological mechanism of TCM/Natural drugs, they were uniformly classified as TCM/Natural drugs. Fourthly, it is unfortunate to exclude some excellent clinical trials due to their incomplete registration information. The small number of such clinical trials did not affect the overall results of this study.

## 5 Conclusion

The study examined the landscape of diabetes drug clinical trials in mainland China between 2013 and 2023 in detail. In the past decade, thanks to advancements in R&D and support from the Chinese government, diabetes drug clinical trials have developed rapidly. Several innovative drugs have been launched in China, providing more therapeutic options for diabetics. Research institutions and pharmaceutical companies need to pay more attention to areas of overlap and make greater efforts to enhance the level of R&D. In the next phase of our plan, we will explore the status of global clinical trials for diabetes medications and conduct a comparative analysis between domestic and international findings.

## Data Availability

The original contributions presented in the study are included in the article/**Supplementary Material**. Further inquiries can be directed to the corresponding authors.
