# Supplementary material for: From past to present: tracing the trends of diabetes drug trials in mainland China
Source: Front Endocrinol (Lausanne). 2025 Jan 10;15:1427148. doi: 10.3389/fendo.2024.1427148 (PMC11757101; doi:10.3389/fendo.2024.1427148)
Supplement: Supplementary file 1 [file Table1.docx]

Supplementary Material

# Supplementary Table

**SUPPLYMENTARY TABLE 1.** Pharmacological mechanism of trial drugs on diabetes

| **Category** | | **Drug(number)** | **Number of drug** | **Number of clinical trials** |
| --- | --- | --- | --- | --- |
| Single agent | DPP-4i | Sitagliptin (46), Vildagliptin (37), Retagliptin (24), Alogliptin (20), Linagliptin (18), Fotagliptin (14), Prusogliptin (14), Trelagliptin (13), Saxagliptin (11),Cetagliptin(10), Yogliptin(9), HSK7653 (9), Teneligliptin(5), Evogliptin (4), TQ05510 (2), Bogliptin(2), Singletine(2), HD118(1), HL012MA(1), TQ-F3083(1), Dorzagliatin(1), Gemigliptin(1), Anagliptin(1) | 23 | 245 |
|  | GLP-1Ra | Semaglutide(27), Liraglutide(24), Exenatide(18), TG103(8), Loxenatide(7), GMA102(6), Ecnoglutide(6), Recombinant Insulin Secretagogue(5),Albenatide(5),Supaglutide(5), Noiiglutide(4), Dulaglutide(4), JY09(4), Recombinant GLP-1 Receptor Agonist Injection(4)， Efpeglenatide(3), GZR18(3), HRS-7535(3), Orforglipron(3), TTP273(3), PB-119(2), HL08(2), Exendin-4-Fc fusion protein injection(2), LY05008(2), SL209(2), BGM0504(2), HSK34890(2), VCT220(2), 14028(1), Lixisenatide(1), BPI-3016(1), Beinaglutide(1), MW04(1), SAL015(1), PF-06882961(1), HB1085(1), SAL0112(1),CJC-1134-PC(1), AP026(1), RAY1225(1), HDM1002(1), ZT002(1), KN056(1), SHR-3167(1), BPYT-01(1),THDBH110(1) | 45 | 176 |
|  | SGLT2i | Empagliflozin(54), Dapagliflozin(37), Henagliflozein(22), Canagliflozin(20), Rongliflozin(10), Janagliflozin(8),Tianagliflozin(4), LH-1801(3), Ertugliflozin(2), Bexagliflozin(2), YL-10069(1), DWP16001(1) | 12 | 164 |
|  | Biguanide | Metformin (135) | 1 | 135 |
|  | SU | Gliclazide(29), Glipizide(26), Repaglinide(18), Glimepiride(17), Mitiglinide(14), Gliquidone(10), Nateglinide(5) | 7 | 119 |
|  | Insulin | Insulin degludec(17), Insulin aspart(5), Insulin glargine(5), Recombinant Human Insulin(4), INS068(4), LY900014(3), THDB0206(3), Isophane Protamine Recombinant Human Insulin(2), LY2963016(2), LY3209590(2), CA001(1) | 11 | 48 |
|  | AGi | Acarbose(38), Miglitol(4) | 2 | 42 |
|  | GKA | Dorzagliatin(11), PB-201(3), Globalagliatin(5), Dorzagliatin(1) | 4 | 20 |
|  | TCM/Nature drug | Ramulus Mori alkaloids tablets(3), Zhi Mu Duo Fen Capsule(1), Jin Li Tang Ping Tablet(1), Jin Qi Jiang Tang Capsule(1), Hu Huang Jiang Tang Capsule(1), Chi Gan Capsule(1), Shan Zhuyu terpene tablets(1), Shu Tang granule(1), San Ye Tang Zhi Tablet(1), Tang Mai Jiao Tai Capsule(1), Zi Chun Tablet(1), Lian Mei Granule(1), Berberine(1) | 13 | 15 |
|  | GIP/GLP-1Ra | Tirzepatide(6), HRS9531(3), HS-20094(2), HZ010(1), HZ012(1), THDBH120(1) | 6 | 14 |
|  | PPAR-γa | Pioglitazone(12) | 1 | 12 |
|  | PPARa | Chiglitazar(10) | 1 | 10 |
|  | GCG/GLP-1RA | Mazdutide(7) | 1 | 7 |
|  | SGLT1i | SY-008(7) | 1 | 7 |
|  | FXRa | HTD1801(6) | 1 | 6 |
|  | PPAR-α/γa | Aleglitazar(5) | 1 | 5 |
|  | GPRR40a | Fuglifam(2), TAK-875(1), TSL-1806(1) | 3 | 4 |
|  | SGLT1/2i | Sotagliflozin(4) | 1 | 4 |
|  | CALCRa | Pramlintide(3) | 1 | 3 |
|  | DRDsa | Bromocriptine(2) | 1 | 2 |
|  | GCGRa | ISIS449884(2) | 1 | 2 |
|  | GCGR/GIPR/GLP-1Ra | UBT251(2), MWN101(1) | 2 | 3 |
|  | GIPRa | NNC0480-0389(1) | 1 | 1 |
|  | FGF21 /GLP-1Ra | HEC88473(2) | 1 | 2 |
| Compound drug | DPP-4i and Biguanide | Sitagliptin/Metformin(46), Linagliptin/Metformin(10),Vildagliptin/Metformin(10), Saxagliptin/Metformin(6), Alogliptin/Metformin(1), Retagliptin/Metformin(1) | 6 | 74 |
|  | SGLT2i and Biguanide | Empagliflozin/Metformin(41), Dapagliflozin/Metformin(41),Canagliflozin/Metformin(4), Henagliflozein/Metformin(2) | 4 | 64 |
|  | PPAR-γa and Biguanide | Pioglitazone/Metformin(18) | 1 | 18 |
|  | SU and Biguanide | Glipizide/Metformin(9), Glyburide/Metformin(2), Glimepiride/Metformin(2), Repaglinide/Metformin(2) | 4 | 15 |
|  | Insulin type combinations | Insulin Degludec/Insulin Aspart(6), Insulin Aspart Protamine/Insulin Aspart(2), Insulin Isophane Human/Insulin Human(5), Insulin Lispro Protamine/Insulin Lispro Injectable Suspension(1) | 4 | 14 |
|  | Insulin and GLP-1Ra | Insulin Glargine/Lixisenatide(4), Insulin Degludec/Liraglutide(4), HR17031(3), Insulin Icodec/Semaglutide(1) | 4 | 12 |
|  | PPAR-γa and SU | Pioglitazone/Glimepiride(9) | 1 | 10 |
|  | DPP-4i and SGLT1/2i | Empagliflozin/Linagliptin(1) | 1 | 1 |
|  | SGLT2i and DPP-4i | THDBH101(1) | 1 | 1 |
|  | AGi and Biguanide | Acarbose/Metformin(1) | 1 | 1 |
